# Exploring DNA methylation profiles in blood samples of canine gastrointestinal lymphoma

**DOI:** 10.1371/journal.pone.0339388

**Published:** 2025-12-30

**Authors:** Miyuki Nakamura, Yuki Matsumoto, Masatoshi Nagata, Keiji Yasuda, Kei Yonekawa, Shigeki Muramatsu, Anna Goshima, Ryo Nakaki, Nozomu Yokoyama, Ko Nakashima, Masahiro Okumura, Jumpei Yamazaki

**Affiliations:** 1 KDDI Research, Inc., Saitama, Japan; 2 Research and Development Section, Anicom Specialty Medical Institute Inc., Kanagawa, Japan; 3 Data Science Center, Azabu University, Kanagawa, Japan; 4 Rhelixa Inc., Tokyo, Japan; 5 Department of Veterinary Clinical Sciences, Laboratory of Veterinary Internal Medicine, Graduate School of Veterinary Medicine, Hokkaido University, Sapporo, Japan; 6 Japan Small Animal Medical Center, Tokorozawa, Saitama, Japan; 7 Laboratory of Veterinary Surgery, Department of Veterinary Clinical Sciences, Graduate School of Veterinary Medicine, Hokkaido University, Hokkaido, Japan; 8 Veterinary Teaching Hospital, Graduate School of Veterinary Medicine, Hokkaido University, Sapporo, Japan; 9 One Health Research Center, Cancer Research Unit, Hokkaido University, Sapporo, Japan; Hazara University, PAKISTAN

## Abstract

Blood-based testing represents a valuable tool for the detection and monitoring of patient conditions in both human and veterinary medicine. When conventional tissue-based diagnosis is challenging, blood-derived measurements allow for minimally invasive testing. Recent studies across mammalian species, particularly in humans, have explored the use of DNA methylation from whole blood, revealing its potential to predict individual mortality and responses to environmental stresses. While it is well recognized that tumor lesions display altered epigenetic modifications across some mammalian species, little is known about how DNA methylation in blood, as an indirect tissue sample, reflects the status of individuals in dogs. In this study, we conducted whole genome bisulfite sequencing using whole blood samples from twenty dogs diagnosed with canine gastrointestinal lymphoma, which is a prevalent disease in dogs. Comparative analysis with non-lymphoma controls identified over one thousand differentially methylated regions (DMRs). To develop practical predictive models, we narrowed down the number of DMRs from the total identified to a feasible set of probes using machine learning, achieving high accuracy (0.8–0.9) in predicting lymphoma cases. Our research underscores the potential of utilizing DNA methylation from whole blood as predictors and establishes a foundational data infrastructure for genome-wide DNA methylation for canine health monitoring for future studies.

## Introduction

DNA methylation is a dynamic epigenetic mark that can reflect various biological processes through extrinsic and intrinsic factors. It is frequently observed on the cytosine of CpG di-nucleotides. This modification is variable based on tissues/cell-types, aging, environmental exposures, and other factors [1]. Recently, there are several attempts to predict individual health status from DNA methylation pattern to support disease diagnosis or monitoring prognosis as a biomarker in human [2–4]. DNA methylation is a covalent bond and is relatively stable and resistant toward degeneration. Moreover, DNA methylation pattern is detectable from small volume of blood [5]. Therefore, DNA methylation biomarkers derived from blood cells are expected to be non-invasive or minimally invasive marker. For example, in human studies, it has been reported that DNA methylation patterns of blood are associated with cancer incidence and mortality [6,7], and post-COVID-19 conditions [8]. In other cases, DNA methylation is also used as a more direct approach to distinguish cancer from normal tissues or to identify the localization of the lesion using cell-free DNA (cfDNA), which includes circulating tumor DNA that is released from affected tissue into blood plasma in those patients [9–13].

In this study, we attempted to predict canine gastrointestinal lymphoma incidence using DNA methylation patterns of whole blood cells rather than addressing cfDNA or tumor lesions. It is sometimes difficult to detect lesions in the cases of lymphoma, although the subject has a suspicion of lymphoma. This complicates the biopsy procedures, underscoring the fundamental requirement for developing of blood markers for diagnostic support. To discover genomic regions that show differences in DNA methylation between lymphoma cases and controls, we performed whole genome bisulfite sequencing (WGBS) and identified nearly 1,500 differentially methylated regions (DMRs) between blood samples from lymphoma and non-lymphoma subjects. To design a cost-effective molecular assay, we defined these DMRs as candidate predictors and narrowed them down to practical set size using machine learning approaches. Our study revealed that the models using a combination multiple DMRs were as effective as those using a single DMR. This study not only highlights the potential of DNA methylation signatures for canine gastrointestinal lymphoma diagnosis but also emphasizes the potential of machine learning in refining predictive models.

## Materials and methods

### Animal and sample preparation

A total of 20 dogs (Canis lupus familiaris) with a confirmed diagnosis of gastrointestinal lymphoma were included in this study. Materials were obtained from two research institutes, Hokkaido University Veterinary Teaching Hospital and Anicom Specialty Medical Institute, Inc., with written consent from the owners for their animals’ participation in this study. This study has been approved by the Laboratory Animal Experimentation Committee of the Graduate School of Veterinary Medicine, Hokkaido University (Approval number: 2022−012) and by the Ethics Screening Committees of Anicom Specialty Medical Institute, Inc. (Approval number: 2022−02), by providing written consent that follows the guidelines. Amount of 0.1–0.4 ml whole blood from each individual was used for DNA isolation. Genomic DNA was isolated from whole blood using the DNeasy Blood and Tissue Kit (QIAGEN, Germantown, MD, USA) following the manufacturer’s protocol. All efforts were made to minimize animal suffering during sample collection.

### Study design and characteristics of subject population

To explore the differentially methylated region, we performed WGBS on blood samples from 20 individual dogs that were diagnosed with canine gastrointestinal lymphoma. As control samples, we retrieved WGBS data from 19 individuals that were nominally healthy group except for injuries in some cases from the previous study (GSE252908) [14]. All samples (both cases and controls) were collected prospectively as part of a single case-control study. Although all measurements were conducted simultaneously, samples originated from multiple facilities, herein referred to as collection setting. A potential limitation of our study is the institutional heterogeneity in sample collection. None the less, the majority of samples (>65%) from both case and control groups were collected at a single facility, which reduces the likelihood of substantial inter-site technical variation. The demographics of subjects in this study was summarized in Table 1.

**Table 1:** Demographics of dog individuals in this study.

	Control (n = 19)	Cases (n = 20)
**Age in years**		
Mean (SD)	7.79 (SD: 3.66)	10.2 (SD: 2.02)
min-max	3 - 14	6 - 15
**Sex**		
Female (Spayed)	13 (5)	10 (7)
Male (Castrated)	6 (5)	10 (7)
**Breeds**		
Bichon frize	–	1
Boston terrier	–	1
Cavalier	–	1
Chihuahua	–	1
Dachshund (Miniature)	3	1
French bulldog	–	1
Golden retriever	–	1
Norfolk terrier	–	1
Papillon	–	1
Pug	–	2
Shetland sheepdog	–	1
Shiba	3	4
Poodle (Standard)	–	1
Poodle (Toy)	13	2
Yorkshire terrier	–	1

### Sequencing and mapping

Genomic DNA extracted from whole blood was sent to Novogene for bisulfite conversion, library preparation, and sequencing. Bisulfite conversion was conducted using EZ DNA Methylation Gold Kit (ZYMO Research, USA). For the library preparation, the Scale Methyl-DNA Lib Prep Kit (ABclonal, China) was used. Prepared libraries for WGBS were sequenced with 150 cycle paired end mode in the NovaSeq. The amounts of reads were targeted to provide 70 × coverage of depth for each subject. Obtained read information was summarized in Tables 2 and 3.

**Table 2:** Information of read counts and mapping statistics from whole genome bisulfite sequencing.

Sample ID	sequenced reads	Obtained data (Gbp)	Mapped read counts	Mapping rate (%)	Counts after removing duplications	Duplicated propotion (%)	Conversion rate
D15	1,471,119,044	220.7	942,210,058	64.05	921,179,226	2.2	0.99
D16	1,374,040,624	206.1	880,626,330	64.09	846,436,780	3.9	0.99
D18	1,621,119,906	243.2	986,042,610	60.82	921,278,317	6.6	0.99
D19	1,488,384,704	223.3	599,523,524	40.28	498,068,081	16.9	0.99
D20	1,335,235,072	200.3	888,439,718	66.54	872,186,632	1.8	0.99
D21	1,504,685,636	225.7	609,850,737	40.53	506,837,326	16.9	0.99
D22	1,440,045,372	216.0	650,928,388	45.20	557,306,630	14.4	0.99
D23	1,661,628,566	249.2	752,672,493	45.30	624,967,698	17.0	0.99
D24	1,458,733,410	218.8	583,552,483	40.00	478,893,383	17.9	0.99
D25	1,630,311,074	244.5	658,822,764	40.41	533,411,778	19.0	0.99
D28	1,432,433,688	214.9	915,182,746	63.89	898,509,744	1.8	0.99
D29	1,360,933,072	204.1	889,512,076	65.36	871,747,385	2.0	0.99
D30	1,370,910,340	205.6	887,239,964	64.72	869,797,915	2.0	0.99
D31	1,431,411,718	214.7	897,085,424	62.67	879,039,244	2.0	0.99
D32	1,311,901,762	196.8	851,540,570	64.91	837,617,920	1.6	0.99
D36	1,442,345,274	216.4	921,197,632	63.87	901,449,651	2.1	0.99
D37	1,470,580,534	220.6	887,838,444	60.37	868,419,348	2.2	0.99
D38	1,505,734,980	225.9	942,032,752	62.56	923,800,492	1.9	0.99
D39	1,582,775,108	237.4	981,349,414	62.00	960,294,884	2.1	0.99
D40	1,446,427,318	217.0	897,388,910	62.04	881,112,307	1.8	0.99

**Table 3:** Summary of sequencing data non-lymphoma and lymphoma subjects.

	Sequenced reads in total	Average of obtained data (Gbp)	Average of mapped read counts	Average of mapping rates (%)	Average of counts after removing duplications	Average of duplicated propotions (%)	Average of conversion rates
Non-lymphoma*	1,464,195,532	219.6	934,960,229.9	64.0	916,300,128.8	2.0	0.99
Lymphoma	1,467,037,860	220.1	831,151,851.9	57.0	782,617,737.1	6.8	0.99

*Non-lymphoma data was retrieved from GSE252908

### Basic data analysis

For the sequence quality control and mapping, the procedures were described in the previous study [14]. In brief, quality filtered reads were mapped to the dog reference genome Canfam3.1 and methylation rate were called using methylpy (v1.2.9) [15]. CpGs with low coverage of depth (less than 10 reads per CpG across all samples) or with extremely high coverage (more than 500 read per CpG in at least one samples) were removed. Approximately 14.2 million CpGs (14,242,200), equal to more than half of all CpGs in the dog reference genome, were analyzed in subsequent analyses. Principal component analysis PCA was conducted using prcomp function in R with scaling option.

### Estimating the variance of the covariates

To estimate the amount of potential factors as covariates in this experiments, Principal variance component analysis (PVCA) was performed using pvca package (v1.50.0) in R with threshold: 0.6 [16]. Potential confounding factors as follows were considered; “breeds”, “age”, “sex”, and “collection setting (sample collection)” in all samples (n = 39).

### Differentially methylated cytosine (DMC) selection

To divide the dataset for training and test sets, 28 individuals were designated as the training set, while the remaining 11 individuals were utilized as the test set. Lymphoma-associated DMCs were called using methylKit (v1.34.0) [17]. Dog breeds and ages of individual cases were included as covariates into the model, with a difference in beta values greater than 10% and q.value (adjusted *p-*value) < 0.05. Furthermore, in order to reduce false positives and identify more reliable DMCs, the non-lymphoma and lymphoma labels were randomly shuffled, and the statistics for each CpG were computed. After one hundred iterations, CpG sites detected more than once even with the permutated labels were subsequently removed from the DMC candidates.

### DMR identification and clustering

DMCs located within 100 bp were combined, and the combined genomic regions including more than 5 DMCs were considered as DMRs. DMRs were visualized using heatmap.2 [18] and annotations were visualized using pheatmap [19]. 1,532 DMRs were clustered using k-means clustering. Prior to the k-means analysis, the optimal number of clusters was determined using the elbow method, which calculates the within-cluster sum of squares (WCSS) in a range of simulated cluster number (S2 Fig in S1 File).

### Gene ontology enrichment analysis

Gene annotations associated with gene ontology and KEGG pathways were acquired through R packages in the following manner: “org.Cf.eg.db” by Carlson M (2022). For KEGG pathway enrichment analysis, all annotated genes (n ≈ 17,000) were used as the background gene set, as implemented in the R package “org.Cf.e.g.,db” (version 3.14.0). WGBS coverage was highly uniform across the genome, with 99.5% of genes containing at least one CpG site with sufficient coverage either within the gene body or within 10 kb of the gene boundaries. Only a small subset of genes (<1%) lacked CpG coverage in these regions, indicating minimal potential bias from uneven sequencing coverage. For Gene Ontology enrichment analysis, we utilized annotations from the “goa_dog_isoform.gaf” (version 108), which contained approximately 8,650 protein annotations [20,21]. Of these, 8,102 proteins were successfully mapped to the reference genome, and 8,080 genes (93.4%) had at least one CpG site with available coverage within 10 kb. These genes were used as the background gene set for Gene Ontology analyses. In order to identify overrepresented terms within the GO analysis, *p-*values were computed with the hypergeometric test and subsequently adjusted by controlling for the false discovery rate. DMR-gene associations were determined using bedtools (v2.30.0) to identify overlapping DMRs with gene bodies and to map intergenic DMRs to their closest genes.

### Simple logistic regression model

The training dataset was used to develop a prediction model, while the remaining data were reserved for evaluating the constructed models. For constructing logistic regression models, we assessed the relationship between average DNA methylation across each DMR and the lymphoma/non-lymphoma status. The predicted status was modeled based on the logit transformation of the probability of having gastrointestinal lymphoma. This process was conducted for each DMR using the `lm` function in R. For a preliminary model selection step, 1,000 bootstrap samples were conducted and then the averagearea under the receiver operating characteristic curve (AUC-ROC) was calculated on the training dataset. To assess the model performance, the AU-ROC and the Area Under the Precision-Recall curve (AU-PRC) were adopted for the evaluation on the test dataset.

### Prediction model construction by machine learning

For multiple logistic regression with regularization, to select informative DMRs at one step, multiple linear regression was performed to construct prediction models. To handle the multicollinearity and relatively large number of features with feasible computing resources, the FastSparse (v0.1.0) package was utilized [22]. The parameter gamma was fixed to 0.001. The best lambda was determined by 4-fold cross-validation on the training dataset. For decision tree and random forest, to select representative DMRs for distinguishing between lymphoma and non-lymphoma individuals, decision trees were constructed for each cluster and the five DMRs in total were selected. Among these selected five DMRs, the lymphoma status was predicted using random forest method.

## Results

### Entire pattern of DNA methylation between subjects with and without a diagnosis of canine gastrointestinal lymphoma

A total of 39 dog individuals (control:19, case:20) were enrolled from participating veterinary hospital and related facilities. Cases were dogs with veterinary-confirmed diagnosis of canine gastrointestinal lymphoma, while controls were dogs that showed no observed internal medical diseases at the time of sample collection. The mean age in years was 7.79 (standard deviation (SD): 3.66) in control, 10.2 (SD: 2.02) in case, respectively. The sex ratio of the population was female (spayed): male (castrated) was 13 (5): 6 (5) in control, 10 (7): 10 (7) in case. In terms of dog breeds, control and case populations consisted of three and fifteen breeds, respectively (Table 1).

**Fig 1:**
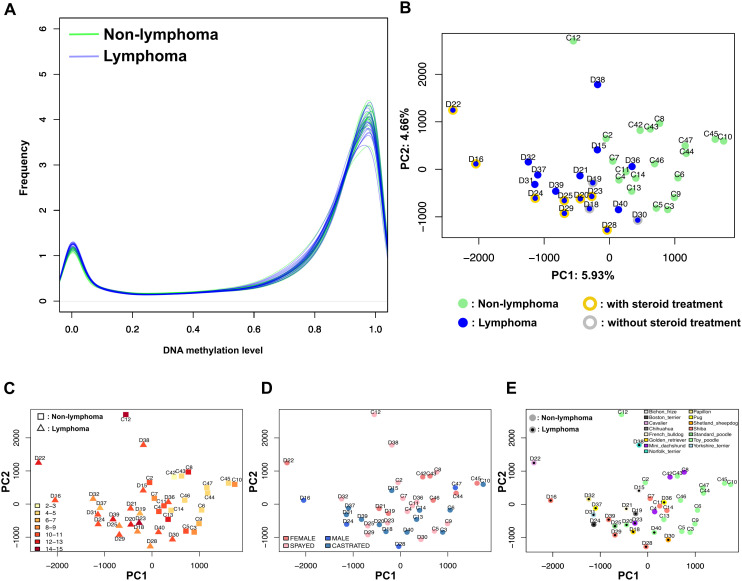
DNA methylation level and the projections of potential covariate attributions. (A) Density plot of DNA methylation level of measured CpGs in each individual. (B) Plot of principal component analysis using all CpGs passing filtering criteria. (C-E) Projections of demographic characters; age (C), sex (D), breeds (E) on the PCA plot.

Quality filtering yielded approximately 14.2 million CpGs for analysis, representing more than half of all CpGs in the dog reference genome. The distribution of the beta values was similar between lymphoma and non-lymphoma individuals (Fig 1A). Next, to investigate the variance of DNA methylation associated with known demographic factors, such as age, sex, breeds, and the diagnosis of lymphoma, we conducted principal component analysis (PCA) using all available CpG except CpG with the same methylation level across all samples. After scaling, lymphoma and non-lymphoma individuals were relatively separated along the first principal component (PC1) (Fig 1B). PC1 and the second principal component (PC2) explained only 5.93% and 4.55% of variance, respectively.

Next, because some subjects had undergone treatment, such as corticosteroids, which are a major treatment for common inflammation. Steroid treatment can affect for blood cell composition [23]. To assess the effect of the steroid treatment, we chose individuals with traceable clinical histories and projected the steroid treatment condition onto the PCA plot (Fig 1B). Although individuals with steroid treatment were located relatively on the right side of the PCA plot, the separation between individuals with and without steroid treatment was not clear. Thus, steroid treatment had no obvious correlation with the high-variance components of DNA methylation.

It is known that DNA methylation is affected by age and sex [24]. To investigate whether age, sex, and dog breeds explained these PC1 and PC2, we projected these three attributions on the PCA plots (Fig 1C-E). Age and sex had no obvious association with either PC1 or PC2. This is because the non-lymphoma group contains a relatively large number of poodle individuals. It appears that poodle breeds form a cluster. However, the same breed such as poodle or shiba were not clustered together over cases and controls. That is, breeds attribution also did not show a strong association with either PC1 or PC2. Thus, several attributes, such as steroid treatment, sex, age, breeds, were not strongly manifested at least along PC1 and PC2. On the other hand, cases and controls were relatively separated, despite the fact the PC1 and PC2 loading explained only approximately 10–11% of the total variance. Additionally, to estimate the contribution of each covariates, we performed Principal variance component analysis (PVCA). PVCA revealed sample collection setting explained only 6.4% of variance, while biological covariates contributed with limited contribution (age: 1.5%, sex: 3.4%, breed: 1.1%) (S1 Fig in S1 File). The substantial residual variance (62.1%) likely encompasses individual variation and disease-associated signals. These findings demonstrate that technical factors have minimal impact on the observed case-control separation, supporting the validity of our methylation profiling results.

### Identification of differentially methylated regions

**Fig 2:**
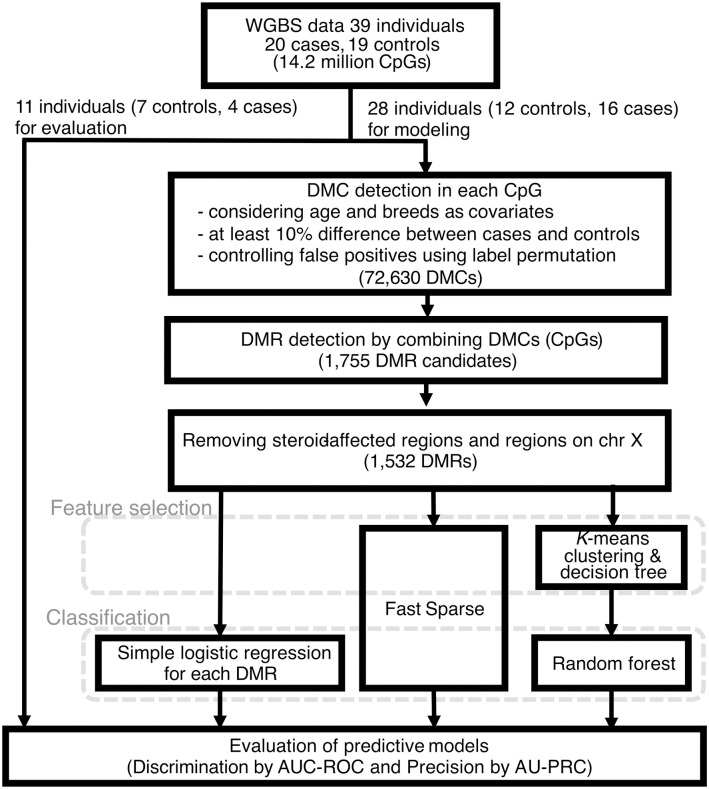
A flow diagram illustrating procedures for construction of the model to predict lymphoma status. The dataset was randomly divided into a training set (approximately 80%) for feature selection and model development, and an independent test set (approximately 20%) for performance validation to ensure unbiased model evaluation. This approach prevents data leakage and ensures an unbiased assessment of model generalizability.

To detect differentially methylated cytosines (DMCs) that compose differentially methylated regions (DMRs), we conducted regression analysis of DNA methylation level in all each qualified CpG site against the diagnosis of canine gastrointestinal lymphoma using the R package methylKit. For predictive modeling, the 39 individuals were divided into a training dataset (28 individuals: 12 controls, 16 cases) and a test dataset (11 individuals: 7 controls, 4 cases) using a hold-out method (S1 Table in S1 File, Fig 2). This process helps to fairly evaluate constructed predictive models by reducing the risk of data leakage. Then, the training dataset was utilized to identify differentially methylated regions. Although we did not see any obvious association with either 1^st^ or 2^nd^ component, still to ensure the robustness of the subsequent analysis, these affect yearly ages and breeds were included as covariate for adjustment to consider the potential confound factors. Moreover, here, to control for false positive signals, the labels of case/control status were permutated 100 times, and DMC candidates that were called more than once, even under the permuted labels, were removed. This process would reduce false positive even using small sample size. Hypomethylated-DMC candidates (156,699) were reduced to 63,455 DMCs and hypermethylated DMC candidates (79,197) were reduced to 9,175, resulting in 72,630 DMCs in total. The remaining DMCs were merged if they were located within a distance of 100 bp. This process identified approximately 1,800 (1,755) regions as lymphoma-DMR candidate regions.

**Fig 3:**
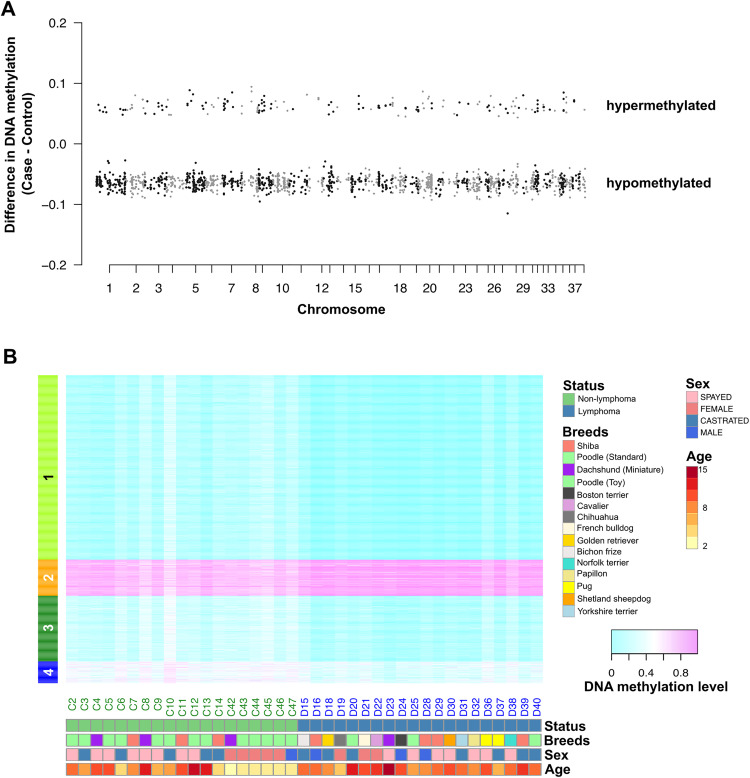
Identification of differentially methylated regions (DMRs) between subjects with and without lymphoma. **(A)** Locations of lymphoma-DMRs across chromosomes. **(B)** Heatmap of average methylation level across identified DMRs by each cluster. Clustering and feature selection were performed on the training set only. The heatmap displays all samples to demonstrate cluster consistency; however, model evaluation was conducted exclusively on the independent test set.

Furthermore, to exclude the CpGs that could respond to steroid treatment, steroid-DMRs were detected using methylKit without any covariate adjustment. This detection process was performed using loose criteria, which contain more than three steroid-DMCs to comprehensively identify steroid affected regions. 6,594 hypermethylated and 7,947 hypomethylated steroid-DMRs in the group with steroid treatments were detected. These genomic regions were subtracted from the lymphoma-DMR candidate. Moreover, to eliminate the potential impact of sex differences on the reliability of the lymphoma-DMRs, the lymphoma-DMRs located on X chromosome were omitted from subsequent analysis. Then, the remaining lymphoma-DMR candidates numbered 1,532 regions. DMRs distributed across the genome (Fig 3A). The difference in average DNA methylation level on each DMR between cases and controls was relatively small and most of them were around 10% in difference.

To obtain the overview and trends in change pattern of DNA methylation of these DMRs, we performed *k*-mean clustering using the DNA methylation level of lymphoma-DMRs. The number of DMRs in each cluster was as follows; 917 DMRs for cluster_1, 180 DMRs for cluster_2, 326 DMRs for cluster_3, and 109 DMRs for cluster_4. Only cluster_2 showed a trend of hypermethylated in the lymphoma group and all other three clusters showed hypomethylated patterns (Fig 3B). The methylation trends between cluster_1 and cluster_3 were similar, but some DMRs in cluster_3 had larger difference in DNA methylation level compared to that in cluster_1. Thus, hypomethylated DMRs were predominant compared to the number of hypermethylated DMRs. Taken together, these identified DMRs can be used to discriminate between individuals with lymphoma and those without lymphoma.

### Profile of genes adjacent to DMRs

**Fig 4:**
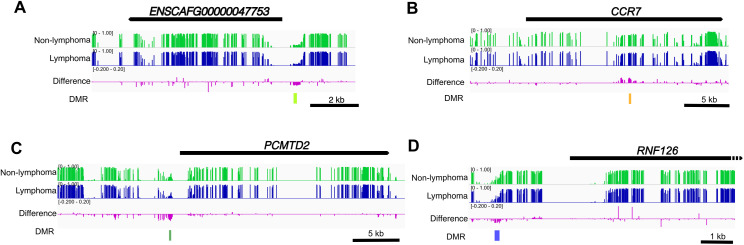
IGV screenshot of representative DMRs from each cluster. **(A-D)** DMRs are shown as rectangle in corresponding colors to the colors of the cluster in Fig 3B; cluster_1 **(A)**, cluster_2 **(B)**, cluster_3 **(C)**, and cluster_4 **(D)**. The height of each bar shows the average of the DNA methylation level on CpG in the control group or the case group.

Among these 1,532 DMRs, 1024 DMRs overlapped gene regions (802 genes), 63 DMRs were located in promoter regions of 59 genes (within 1.5 kb of upstream genes), and 278 DMRs located within 10 kb of at 249 genes (Datasheet S2 File). These DMR adjacent or overlapped genes included those involved in blood cell development and differentiation, such as *CCR7*, *CD27*, *PRDM1*, *GFI1* and *FCMR* in other mammals [25–28]. The DNA methylation status at representative loci was visualized in Fig 4A-D. Enrichment analysis using gene ontology showed a relatively limited number of enriched GO terms. Among genes with DMRs at their promoter regions, only ‘extracellular space (GO:0005615)’ was enriched (S2 Table in S1 File). Among DMR-adjacent genes (located within 10 kb), ‘tumor necrosis factor receptor binding (GO:0005164)’ was enriched (S3 Table in S1 File). For genes overlapped with DMRs, there was no strong enrichment of GO term. Thus, DMR-adjacent genes may be related to immune responses, suggesting a systemic response to pathology.

### Status prediction by single DMRs

To identify potential biomarker candidates, first, we adopted simple logistic regression for each DMR to construct simple models using the training dataset. Next, we applied bootstrapping statistics as the preliminary model selection from 1,532 models constructed on the training dataset and then calculated the average area under the receiver operating characteristic curve (AU-ROC) and the confidential intervals. AU-ROC indicates the ability to discriminate between both positive and negative cases.

The models were evaluated using two metrics: the AU-ROC and the area under the precision-recall curve (AU-PRC). The resultant models were evaluated on the test dataset consisting of 11 individuals, which included 7 subjects without lymphoma and 4 subjects with lymphoma. AU-PRC primarily shows the ability to discriminate among positive cases, making it particularly beneficial for datasets with uneven case/control distributions. Here, we focused on the top twenty DMRs with the highest AU-ROC on the training dataset. These top twenty DMRs contained both hypermethylated and hypomethylated regions (S3 Fig in S1 File). Among them, 14 models based on single DMR perfectly predicted the outcomes with both AU-ROC and AU-PRC equal to 1 (Table 4). However, when these twenty models were tested on the test dataset, these twenty model’s values varied, ranging from 0.643 to 1.000 for AU-ROC and from 0.407 to 1.000 for AU-PRC. Taken together, some simple models consisting of single DMRs had good exhibited predictive power. For example, the model using chr6:15948237–15948396 achieved AU-ROC:1.000 and AU-PRC:1.000 on the test dataset. This model is a high potential to discriminate between case and control. On the other hand, because we assessed a large number of DMRs, we cannot exclude the possibility that the favorable results may be coincidental. While some DMRs showed promising predictive power, it is important to develop robust models to ensure consistent performance across different populations of subjects.

**Table 4:** Top twenty DMRs with high area under the receiver operating characteristic curve and area under the precision-recall curve in simple logistic regression.

DMR Location utilized for the model	Train				Test	
	AU-ROC	AU-PRC	95% CI of AU-ROC in bootstrap	Average AU-ROC of bootstrap	AU-ROC	AU-PRC
chr1:1536481–1536668	1.000	1.000	(-, -)	1.000	0.786	0.675
chr19:53215691–53215832	1.000	1.000	(-, -)	1.000	0.804	0.686
chr20:46145432–46145499	1.000	1.000	(-, -)	1.000	0.804	0.686
chr24:42452432–42452888	1.000	1.000	(-, -)	1.000	0.750	0.758
chr29:28301558–28301659	1.000	1.000	(-, -)	1.000	0.804	0.686
chr3:40825387–40825441	1.000	1.000	(-, -)	1.000	0.732	0.655
chr30:11809493–11809601	1.000	1.000	(-, -)	1.000	0.804	0.686
chr31:26117381–26117527	1.000	1.000	(-, -)	1.000	0.732	0.563
chr6:15948237–15948396	1.000	1.000	(-, -)	1.000	1.000	1.000
chr6:40261667–40261740	1.000	1.000	(-, -)	1.000	0.661	0.480
chr7:65038613–65038736	1.000	1.000	(-, -)	1.000	0.786	0.675
chr9:2603084–2603205	1.000	1.000	(-, -)	1.000	0.839	0.863
chr9:3546369–3546467	1.000	1.000	(-, -)	1.000	0.804	0.686
chr9:58673534–58673663	1.000	1.000	(-, -)	1.000	0.929	0.800
chr19:45357476–45357606	0.995	0.996	(0.922, 1.000)	0.995	0.893	0.884
chr16:46129799–46129902	0.995	0.996	(0.938, 1.000)	0.995	0.821	0.853
chr20:57947794–57948043	0.995	0.996	(0.918, 1.000)	0.995	0.893	0.837
chr22:1692606–1692973	0.995	0.996	(0.918, 1.000)	0.995	0.786	0.781
chr38:20351856–20352012	0.995	0.996	(0.936, 1.000)	0.995	0.643	0.407
chr37:317341–317459	0.995	0.996	(0.895, 1.000)	0.995	0.821	0.853

### Prediction models comprising optimal multiple probes

While a part of simple linear regression models provided accurate predictions with single predictors, DNA methylation levels can be influenced by various intrinsic and extrinsic factors, such as age and environmental stresses. These factors may compromise the reliability of the results. To reflect multiple factors that influence DNA methylation, we constructed predictive models that consist of multiple probes (DMRs as predictors). However, to keep cost manageable, it is important to limit the number of probes used. This approach allows for targeted analysis without the high expenses of genome-wide approach; for example, collecting data from several targeted loci using PCR or locus-specific sequencing is significantly cheaper than a genome-wide approach. Given these considerations, we aimed to narrow down the number of probes to a practical level. Although predictive models with more probes tend to provide more information, the cost of examining the models makes it advantageous to use smaller subsets of probes. This approach not only facilitates practical applications but also helps avoid overfitting. Here, to select effective probes and construct prediction models, we used two different machine learning approaches and compared their performances; 1) multi-linear regression with regularization and 2) feature selection based on heuristics, utilizing decision tree that can reflect potential non-linear structure.

**Fig 5:**
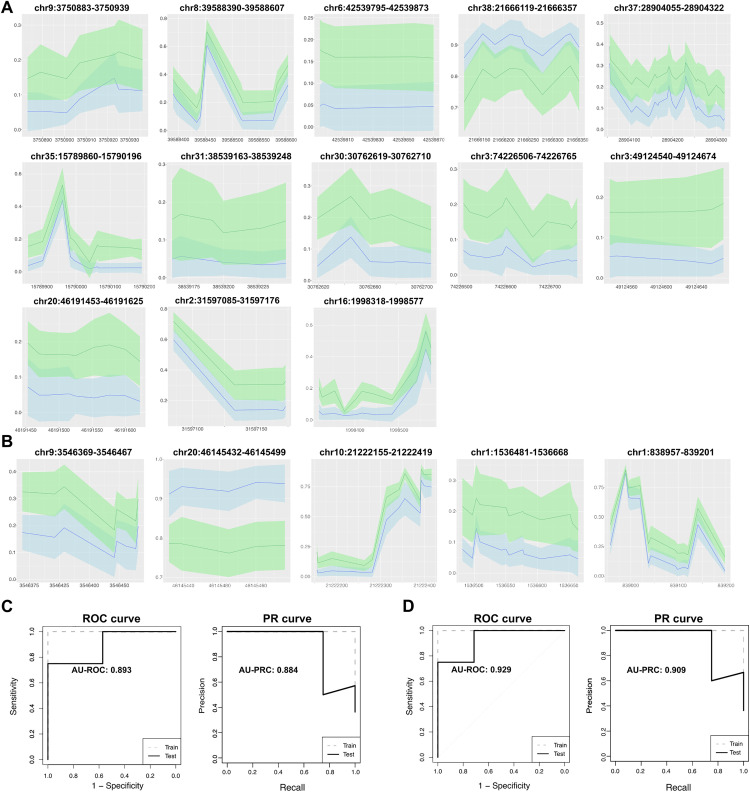
Prediction models of lymphoma consist of multiple probes and their evaluations. **(A, B)** Shaded line plot across DMR regions identified by FastSparse **(A)** (model 1) and by decision tree from each cluster **(B)** (model 2). Green lines indicate the means of the DNA methylation level in controls. Blue lines indicate the means of that in cases. Shaded regions around lines indicates standard deviations. Means and standard deviations were calculated based on the total of the training dataset and the test dataset for the purpose of visualization. **(C-D)** ROC Curve and the precision-recall curve for model 1 (C) and model 2 **(D)**.

First, we adopted a model using multiple logistic regression with regularization. Typically, the multiple regression approach suffers from multicollinearity; in particular, the DNA methylation level also showed similarities between different DMRs (Fig 3B). We adopted the Fast Sparse approach, which can overcome multicollinearity and achieve feature selection using L0 and L2 regularization. L0 and L2 regularization is advantageous for high-dimension data, such as genome-wide data. Using 4-fold cross-validation, we determined the best value of hyper-parameter lambda for regularization (model 1). This method selected 13 DMRs, and the evaluation of this model yielded AU-ROC: 0.893 and AU-PRC: 0.884 on the test dataset (Fig 5A, C).

Turning to a different approach, we also tried feature selection based on the results of k-means clustering (Fig 3B). In this approach, we performed feature selection using decision tree methods in each cluster to avoid multicollinearity. We adapted selected DMRs for an interpretable modeling structure, including non-linear model, to account for potential hierarchical structure, as hierarchical structures were assumed to exist over genomes, such as genetic epistasis. We performed decision tree analyses to choose representative predictors from each of the four clusters of DMRs, reflecting different aspects of the methylation status of the gastrointestinal lymphoma group. This yielded one or two DMRs from each cluster, resulting in a total of 5 DMRs selected. Altering the random seed did not affect the selected subset of DMRs. These DMRs contain both hypermethylated and hypomethylated regions. Furthermore, by utilizing these five DMRs, we performed random forest analysis to model the prediction of having a gastrointestinal lymphoma (model 2). The evaluation of this model yielded an AU-ROC: 0.929 and an AU-PRC: 0.909 on the test dataset (Fig 5B, D). These results suggest that models consisting of multi-probes also discriminate cases from controls relatively well, although the predictions were not perfect.

## Discussions

In this study, we investigated whole genome DNA methylation using whole blood from dogs to distinguish gastrointestinal lymphoma cases from non-lymphoma controls. Unexpectedly, we observed differences in DNA methylation trends between cases and controls (Fig 1). Overall patterns showed a tendency for clustering of each group along PC1 and PC2. However, the boundaries between cases and controls were not clearly defined (Fig 1). These results suggest that DNA methylation from whole blood could be used to monitor the health status of dogs. We obtained 1,532 DMRs from the WGBS analysis (Datasheet S2 File), although the whole blood assessed was an indirect tissue that did not include lesions.

However, the causality of DNA methylation alteration in individuals with canine gastrointestinal lymphoma remains unclear. One possible explanation is that DNA methylation reflects the systemic response to lymphoma. Supporting this hypothesis, some calcium binding proteins tend to be dysregulated in human pan-cancer [29], and two of these proteins were adjacent to DMRs in our dataset. Similarly, several genes adjacent to DMRs in our dataset represent canine homologs of human cancer-associated genes: TNFSF13 is known to be related to tumor cell proliferation in human studies [30], while RUNXs and MPZL2 are associated with leukemia in human [31,32]. In line with this, some immune response-related genes were adjacent to DMRs (Datasheet S2 File). This observation is similar to findings of human studies, where blood DNA methylation signatures are mainly derived from epigenetic change in immune and inflammatory cells [8,33,34]. At least, here, we excluded potentially steroid-affected genomic regions to avoid influencing of steroid treatment on the prediction in this study. However, like many other blood markers, DNA methylation profile may not serve as direct indicators of the disease, but rather as indirect indicators that reflect a variety of factors and conditions, such as gastrointestinal inflammations or other types of tumors. Therefore, it is important to evaluate them in conjunction with other clinical information for an applied assessment.

So far, several studies have reported the DNA methylation status of DMRs between affected tissues with pathological features and normal or surrounding tissues in several different types of canine lymphoma [35–39]. This study contrasts with these conventional approaches by aiming non/low-invasive assay using whole blood samples rather using biopsy tissue samples from lesions. DNA methylation pattern reflects cell composition, which could depend on subject’s health status. Indeed, an increase in leukocyte composition is sometimes observed in lymphoma cases [40]. Due to the lack of reference data on DNA methylation patterns of each cell type in dogs, we could not assess cell composition. While we do not exclude the possibility that the DNA methylation difference derived from alterations in cell composition, it is noteworthy that DNA methylation in whole blood has a signature that can discriminate lymphoma cases from controls, including a potential of cell composition inference. Future research could benefit from considering cell compositions to better understanding of the mechanism of DNA methylation changes.

We also challenged high-dimensional features with only a small number of samples in selecting good predictors from the detected DMRs. Simple logistic regression identified relatively good predictors, although some of the predictors showed poor accuracy on the test dataset. A concern with simple logistic regression models is the presence of random variation during evaluation processes, which may lead some models to yield favorable results on the test dataset purely by chance. This might be caused by the small sample size of the dataset, which can lead to overfitting. For future analyses, it is essential to conduct an independent validation study. To mitigate the risk DNA methylation changes in the specific predictors due to unintended factors, we also designed the model composed of multiple predictors (Fig 5). In the minimum case, five predictors showed relatively good score of gastrointestinal lymphoma prediction. For validation studies and construction of better models, a larger number sample size might improve these models in future. Thus, we selected predictive genomic regions for affected individuals using machine learning approaches and identified promising regions. This data will be leveraged for future panel design, utilizing these data will enable broader applications, such as assessment of disease subtype, tumor stage evaluation, or monitoring of general health status.

## Supporting information

S1 File Supplementary Figures and Tables. **Fig S1.** Principal Variance Component Analysis (PVCA) showing variance components explained by each potential covariate. The analysis was performed to evaluate possible confounding effects of unmodeled variables. **Fig S2.** Total within clusters sum of squared error in different number of clusters (from 2 to 10 clusters). **Fig S3.** Jitter plots of average methylation level in DMRs identified by decision tree from each cluster. **Table S1.** Demographic statistics of dog individuals in the train and the test datasets. **Table S2.** Enrichment analysis of gene ontology (GO) on the genes set having DMRs in their promoter. **Table S3.** Enrichment analysis of gene ontology (GO) on the genes adjacent to DMRs within 10 kbp Data sheet 1 Identified differentially methylated regions and adjacent genes.
(PDF)

S2 File Supplemental_DataSheet1. (CSV)
