# Supplementary material for: Exploring DNA methylation profiles in blood samples of canine gastrointestinal lymphoma
Source: PLoS One. 2025 Dec 30;20(12):e0339388. doi: 10.1371/journal.pone.0339388 (PMC12752990; doi:10.1371/journal.pone.0339388)
Supplement: S1 File — Fig S1. Principal Variance Component Analysis (PVCA) showing variance components explained by each potential covariate. The analysis was performed to evaluate possible confounding effects of unmodeled variables. Fig S2. Total within clusters sum of squared error in different number of clusters (from 2 to 10 clusters). Fig S3. Jitter plots of average methylation level in DMRs identified by decision tree from each cluster. Table S1. Demographic statistics of dog individuals in the train and the test datasets. Table S2. Enrichment analysis of gene ontology (GO) on the genes set having DMRs in their promoter. Table S3. Enrichment analysis of gene ontology (GO) on the genes adjacent to DMRs within 10 kbp Data sheet 1 Identified differentially methylated regions and adjacent genes. (PDF) [file pone.0339388.s001.pdf]

Table S1 Demographic statistics of dog individuals in the train and the test datasets

	Train		Test	
	**Control (n=12)**	**Cases (n=16)**	**Control (n=7)**	**Cases (n=4)**
**Age in years**				
Mean (SD)	7.6 (SD: 3.7)	9.9 (SD: 2.1)	8.1 (SD: 3.9)	11.2 (SD: 1.5)
min-max	3 - 13	4 - 14	6 - 15	10 - 13
**Sex**				
Female(Spayed)	7(4)	8(6)	6(4)	2(1)
Male(Castrated)	5(4)	8(5)	1(1)	2(2)
**Breeds**				
Bichon frize	-	1	-	-
Boston terrier	-	1	-	-
Cavalier	-	-	-	1
Chihuahua	-	1	-	-
Dachshund (Miniature)	2	1	1	-
French bulldog	-	1	-	-
Golden retriever	-	1	-	-
Norfolk terrier	-	-	-	1
Papillon	-	1	-	-
Pug	-	2	-	-
Shetland sheepdog	-	1	-	-
Shiba	2	4	1	-
Poodle (Standard)	-	1	-	-
Poodle (Toy)	8	-	5	-
Yorkshire terrier	-	1	-	2

Table S2 Enrichment analysis of gene ontology (GO) on the genes set having DMRs in their promoter

GO term	GO ID	count in query	count in total	p-values	FDR	Corresponding genes
extracellular space	GO:0005615	2	188	0.022013485	0.04769588	GPI, TNFSF13

Table S3 Enrichment analysis of gene ontology (GO) on the genes adjacent to DMRs within 10 kbp

GO term	GO ID	count in query	count in total	p-values	FDR	Corresponding genes
tumor necrosis factor receptor binding	GO:0005164	3	16	4.38E-05	0.00919014	LTB, TNFSF13, TRAF5

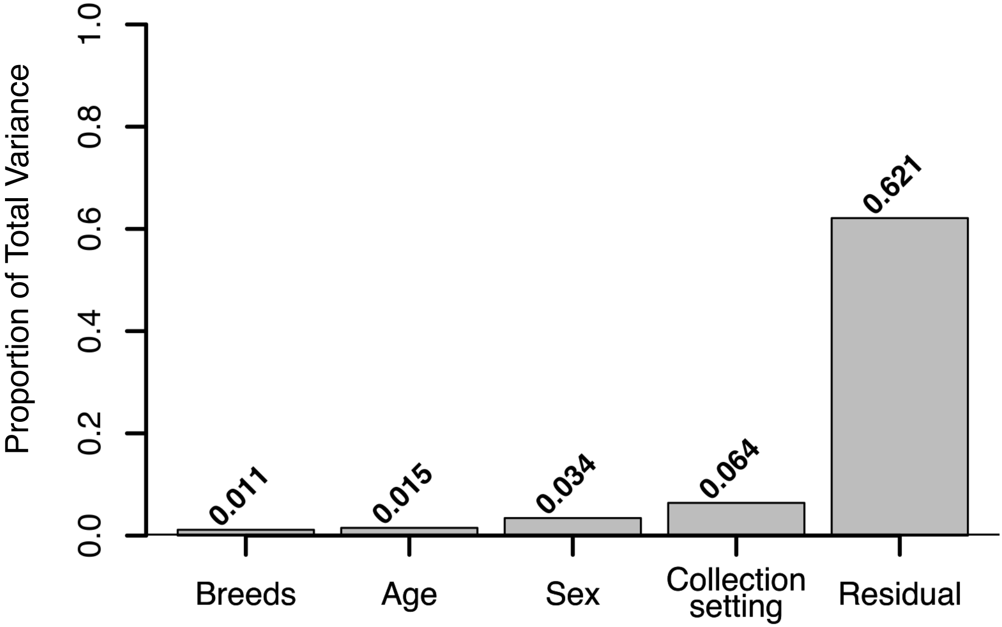

Fig. S1 Principal Variance Component Analysis (PVCA) showing variance components explained by each potential covariate. The analysis was performed to evaluate possible confounding effects of unmodeled variables.

Supplemental Fig.S1

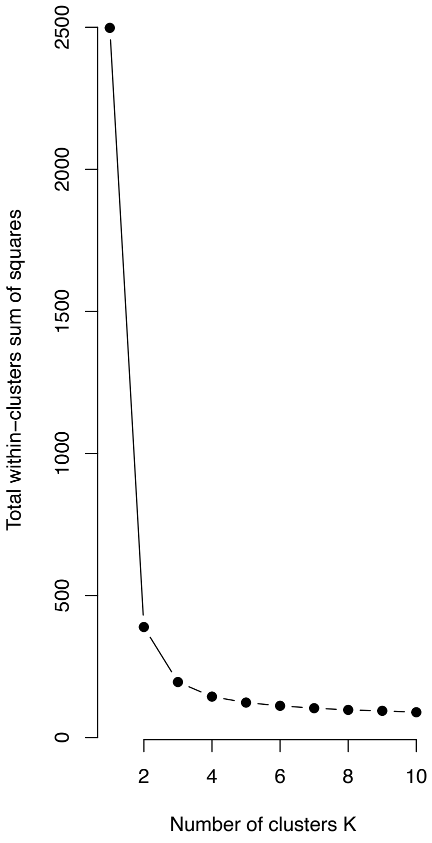

Fig.S2 Total within clusters sum of squared error in different number of clusters (from 2 to 10 clusters).

Supplemental Fig.S2

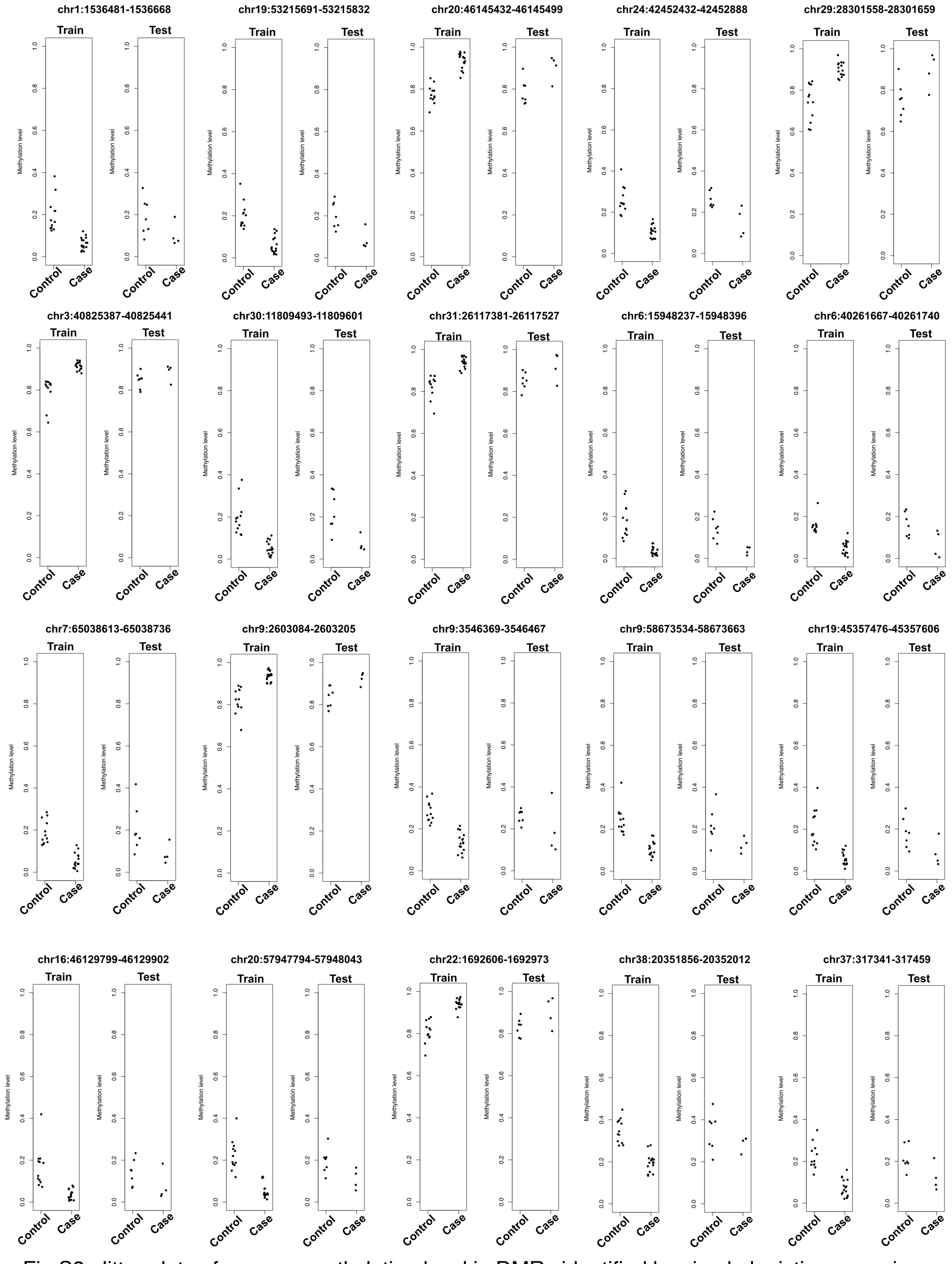

Fig.S3 Jitter plots of average methylation level in DMRs identified by simple logistic regression.

Supplemental Fig.S3
